# Immunology of EBV-Related Lymphoproliferative Disease in HIV-Positive Individuals

**DOI:** 10.3389/fonc.2020.01723

**Published:** 2020-09-30

**Authors:** Polina Shindiapina, Elshafa H. Ahmed, Anna Mozhenkova, Tamrat Abebe, Robert A. Baiocchi

**Affiliations:** ^1^Division of Hematology, Department of Internal Medicine, The Ohio State University, Columbus, OH, United States; ^2^Comprehensive Cancer Center, The James Cancer Hospital and Solove Research Institute, The Ohio State University, Columbus, OH, United States; ^3^Department of Microbiology, Immunology, and Parasitology, School of Medicine Tikur Anbessa Specialized Hospital, College of Health Sciences, Addis Ababa University, Addis Ababa, Ethiopia

**Keywords:** Epstein-Barr virus, lymphoproliferative disease, HIV, Immunology, immunodeficiency

## Abstract

Epstein-Bar virus (EBV) can directly cause lymphoproliferative disease (LPD), including AIDS-defining lymphomas such as Burkitt’s lymphoma and other non-Hodgkin lymphomas (NHL), as well as human immunodeficiency virus (HIV)-related Hodgkin lymphoma (HL). The prevalence of EBV in HL and NHL is elevated in HIV-positive individuals compared with the general population. Rates of incidence of AIDS-defining cancers have been declining in HIV-infected individuals since initiation of combination anti-retroviral therapy (cART) use in 1996. However, HIV-infected persons remain at an increased risk of cancers related to infections with oncogenic viruses. Proposed pathogenic mechanisms of HIV-related cancers include decreased immune surveillance, decreased ability to suppress infection-related oncogenic processes and a state of chronic inflammation marked by alteration of the cytokine profile and expanded numbers of cytotoxic T lymphocytes with down-regulated co-stimulatory molecules and increased expression of markers of senescence in the setting of treated HIV infection. Here we discuss the cooperation of EBV-infected B cell- and environment-associated factors that may contribute to EBV-related lymphomagenesis in HIV-infected individuals. Environment-derived lymphomagenic factors include impaired host adaptive and innate immune surveillance, cytokine dysregulation and a pro-inflammatory state observed in the setting of chronic, cART-treated HIV infection. B cell factors include distinctive EBV latency patterns and host protein expression in HIV-associated LPD, as well as B cell-stimulating factors derived from HIV infection. We review the future directions for expanding therapeutic approaches in targeting the viral and immune components of EBV LPD pathogenesis.

## Introduction

Multiple epidemiologic studies have identified Epstein-Bar virus (EBV)-directed antibodies in more than 90% of the human population (1). The virus causes a life-long latently infected state. In most cases, latent EBV carriers are asymptomatic. However, EBV is associated with lymphoproliferation, and may contribute to the development of lymphoproliferative diseases (EBV-LPD), including B-cell non-Hodgkin lymphomas (NHL), Hodgkin’s lymphoma (HL), NK/T−cell, and peripheral T cell lymphomas (2–6). Individuals with impaired cellular immunity, ranging from inherited immune deficiencies to iatrogenic immunosuppressed state in solid organ and hematopoietic stem cell transplant recipients and individuals infected with the human immunodeficiency virus (HIV), are at a greater risk of developing EBV LPD.

While the risk of developing AIDS-defining lymphomas has diminished from 18% in the pre- combination anti-retroviral therapy (cART) to 4.2% in the post-cART era, the risk of developing HL and NHL showed a lesser magnitude of reduction from 3.9 to 2.2%, based on data acquired from patients between 1980 and 2006 (7). Risk of HL did not reduce with initiation of cART (7, 8). EBV is involved in the pathogenesis up to half of all AIDS-related lymphomas and almost all cases of HIV-associated Hodgkin lymphoma (HL). In this review, we will discuss the mechanisms of EBV-driven lymphomagenesis, the adaptive immune responses to the virus and mechanisms of immune escape. We will summarize the effects of chronic HIV infection on cellular immunity that may contribute to increased risk of EBV LPD in individuals with controlled HIV viremia. Finally, we will touch on the role of immunomodulatory therapies for EBV LPD in HIV-positive [HIV(+)] individuals.

## Mechanisms of EBV Infection and Lymphomagenesis

Epstein-Bar virus infects a variety of human cells, including B, NK, and T lymphocytes and epithelial cells. Initial primary EBV infection occurs through epithelial cells in oropharynx and salivary glands, followed by infection of B cells in the tonsils (9). During the primary infection of B cells, viral glycoprotein (gp) 350 binds to the cluster of difference 21 (CD21, also known as complement receptor II or CR2) located on the cellular surface (10, 11). Almost 100 pre-latent genes are expressed immediately upon primary infection of B cells (12), with several examples shown in Table 1. The immediate-early BamHI ZLF1 gene product BZLF1, also known as Zta, or ZEBRA) is a transcription *trans*-activator homologous to the activating protein 1 (AP-1) transcription factor family (13–16). BZLF1 works in concert with transcriptional activator BRLF1 (17) to initiate expression of a cascade of lytic phase EBV genes including BMRF1 (18–20), BALF1 (21, 22), BHRF1 (23, 24), BALF3 (25), BCRF1 (26), BGLF5 (27), BALF4 (28, 29), BLLF1 (30), and (Table 1), among others, that facilitate the production, maturation, packaging, and release of competent virus particles (31–33). BZLF1 also binds directly to the lytic origin of viral replication and regulates the switch from latent phase of infection to lytic virus production (34, 35). Viral infection contributes to the activation and proliferation of B cells, as marked by increase in the expression of CD40, CD29, and Ki-67 within hours of primary infection (36).

**TABLE 1:** Expression of EBV gene products and their functions during lytic phase of infection.

**Lytic phase stage**	**EBV gene**	**Function of gene product in productive infection**
Immediate early	BZLF-1	Transcriptional activator, essential for lytic infection
	BRLF-1	Transcriptional activator, essential for lytic infection
Early	BMRF-1	Transcriptional activator, viral DNA processivity factor
	BALF-1	Viral Bcl-2 homologue, anti-apoptotic, associates with Bax and Bak
	BHRF-1	Viral Bcl-2 homologue, anti-apoptotic, bocks the pro-apoptotic PUMA factor, inhibition of p53-dependent DNA damage response
	BALF-3	Terminase, involved in DNA synthesis and packaging, required for the production of mature virions
	BCRF-1	Viral interleukin-10 (vIL-10), enhances outgrowth of EBV transformed cells
	BGLF-5	Viral exonuclease, participates in viral linear genome synthesis, viral encapsidation, nuclear egress, virus production
Late	BALF-4	Envelope glycoprotein, required for target cell infection, viral fusion with plasma membrane and endosomal membrane
	BLLF-1	Envelope glycoprotein, initiates virion attachment to host B-lymphocyte cell, leading to virus entry

**FIGURE 1:**
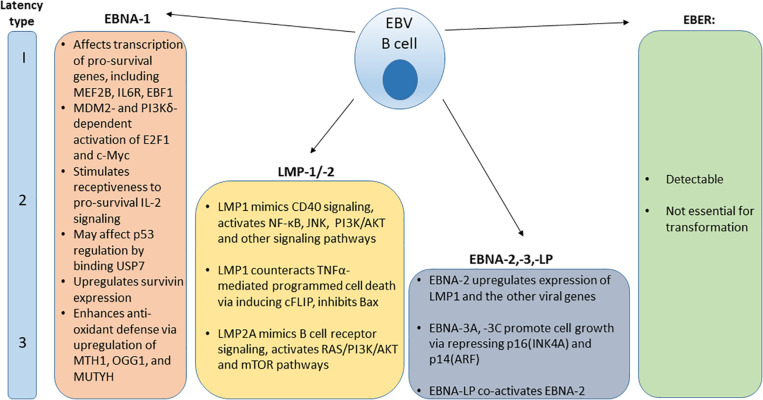
Distribution and function of EBV protein expression and EBER in various viral latency types.

After the initial burst of active viral replication in the oropharynx, EBV establishes a lifelong persistence in latently infected B cells. A population of latently-infected EBV positive memory B cells has been identified in the peripheral blood of IM patients. This indicates that the virus generates a reservoir of persistent latent infection (37, 38). EBV can reactivate to undergo periods of spontaneous lytic replication and virus particle shedding (1). Latently infected B cells persist and may express one of three distinct viral latency patterns, marked by expression of specific viral proteins (Figure 1). Latency type I was initially observed in BL biopsy specimens and BL cell lines and is characterized by the limited expression of the viral nuclear antigen EBNA1 (39, 40). Latency type II can be characterized by the expression of EBNA1 and latent membrane proteins LMP1 and LMP2 and has been observed in EBV-associated nasopharyngeal carcinoma samples and derivative cell lines, HL and some cases of hemophagoytic lymphohistiocytosis (HLH) (41–43). In latency type III, infected B cells express six nuclear antigens (EBNA1, EBNA2, EBNA3A, EBNA3B, EBNA3C, and EBNA-LP), three latent membrane proteins (LMP1, LMP2A, and LMP2B) and transcripts from the miRNA cluster BamHI-A region (BARTs) (44). Type III latency has been observed in post-transplant lymphoproliferative disorder (PTLD) and in lymphoblastoid B cell lines (LCLs) that have been immortalized by EBV viral infection *in vitro* (45). In all latency types, infected cells express two EBV-encoded small RNAs, EBER-1, and EBER-2 (46).

Multiple intracellular signaling pathways (BCR, PI3K/AKT, and CD40) engaged by EBV gene products may contribute to B cell transformation, including lytic and latent viral proteins (47, 48). In addition to aberrant signaling pathways, EBV infection is also associated with acquisition of enhanced mutational burden that may also contribute toward cell transformation. Whole genome sequencing of endemic EBV-associated, HIV-associated and sporadic BL tumors determined that EBV-associated cases had a distinct somatic mutational signature and higher mutational load, compared to EBV-negative cases (49). EBV-associated cases had more frequent mutations in tumorigenic drivers BCL7A and BCL6, suggesting that endemic EBV infection predisposes B cells to specific downstream genetic events as they are transformed (49).

BZLF1 represents an example of lytic viral protein engagement in promoting B cell survival. Wild type BZLF1 is able to enhance proliferation of B cells, unlike BZLF1-knockout EBV (14). BZLF1 directly interacts with the tumor suppressor protein p53 that induces apoptosis in response to DNA damage *in vitro* and *in vivo* (50). BZLF1 promotes the ubiquitination of p53 in the ECS (Elongin B/C-Cul2/5-SOCS-box protein) ubiquitin ligase complex by functioning as an adaptor for p53, and thus targets p53 for degradation (51). Expression of BZLF1 in human T LCLs was associated with decreased NF-κB transcription, decreased expression of an NF-kB reporter gene, and decreased DNA binding by components of NF-κB (52). BZLF1 was also shown to physically interact with the NF-κB p65 subunit when introduced into HeLa cells by expression vector, as well as endogenous p65 in the B cell line Raji, and was able to inhibit BZLF1-driven transcription of viral promoters, leading to the hypothesis that p65 may contribute to maintenance of viral latency (53). Inhibition of nuclear factor kappa B (NF-κB) prevents progression of EBV LPD tumor growth in mice and results in down-regulation of pro-survival genes, assessed by microarray (54).

Latent viral proteins expression also modulate intracellular signaling cascades, leading to cellular immortalization (55). Examples of signaling cascades that EBV latent phase proteins engage in in latently infected and transformed cells are shown in Figure 1, including pro-survival and anti-apoptotic functions of ENBA-1 (56–62), LMP-1/-2 (63–71), and EBNA-2,-3,-LP (72–74) are shown in Figure 1.

## Immune Responses to EBV Infection

**FIGURE 2:**
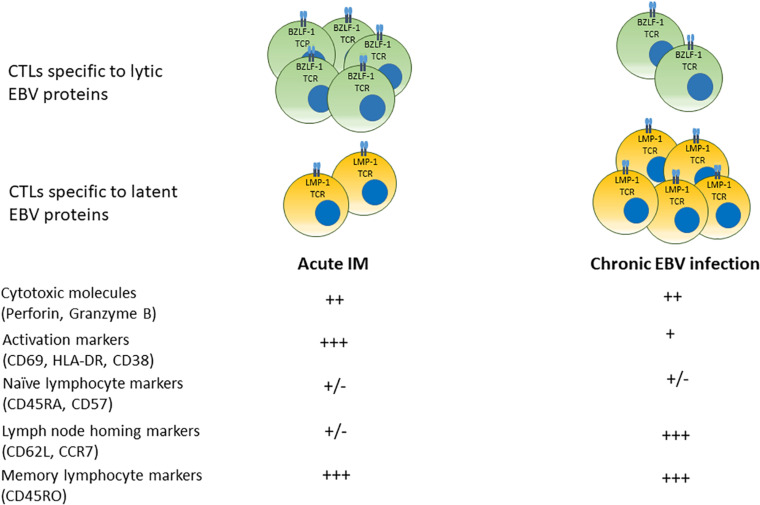
Phenotypes of cytotoxic T cell responses in acute IM and chronic EBV infection. In acute infection, populations of CD8+ T cells specific to (predominantly) lytic EBV antigens bearing markers of activation and memory differentiation rapidly expand in the peripheral blood. Over time, EBV-seropositive convalescent carriers display a prevalence of T cells specific to latent EBV proteins that display a central memory phenotype with greater numbers of lymphocyte homing receptors.

Lytic and growth-transforming latent EBV infection is subject to immune cell-mediated responses (75). Antigen-specific T cell-mediated immune control occurs both during primary infection and throughout life (76, 77). A summary of temporal progression of the specificity and phenotype of circulating cytotoxic T cells specific to lytic and latent EBV antigens over time from acute infection to convalescent state is shown in Figure 2.

Multiple studies suggest that patients with acute IM mount cytotoxic T cell responses, initially to lytic EBV antigens, although responses to latent EBV antigens also arise (78, 79). IM is associated with a profound expansion of CD8+ cytotoxic T cells that are EBV-specific and express markers of activation such as HLA-DR, and cytolytic molecules such as Granzyme B (78, 80, 81). EBV-specific CD8+ T cells were identified by affinity to tetramers of MHC molecules bound to specific EBV peptide epitopes derived from lytic and latent viral proteins (78, 79). Responses to lytic epitopes (BMLF-1, BZLF-1, and BRLF-1) were more prevalent during acute infection, while responses to latent epitopes (EBNA-3A, EBNA-3C, and LMP-1) were more frequently found during latent infection (79). EBV-specific CD8+ T cells were detected in human subjects at least 3 years after the initial infectious mononucleosis (IM) symptoms, indicating that EBV-specific CD8+ T cells responses persist long term after the initial infection (78). Circulating cytotoxic T cells specific to latent EBV antigens were more prevalent in convalescent patients several years after primary infection (78, 79). However, CD8+ T cells with responces to both lytic and latent epitopes derived from BMLF-1, EBNA-3A, LMP-1, LMP-2, and pp65 have been reported in convalescent subjects, indicating the long term prevalence of EBV-specific T cells in latently infected individuals (78, 79, 82, 83). A comparison between responses to immediate early, early and late lytic EBV protein epitopes in activated CD8+ T cells derived from patients with IM and memory CD8+ T cells from patients with chronic EBV showed that patients with IM had most prominent responses to immediate early and early lytic epitopes, while long term EBV carriers have demonstrated highest cytotoxic T cell responces to late lytic viral proteins, suggesting that anti-viral immune responses are modulated in different EBV infection stages (84).

CD8+ T cells displayed surface markers of activation and an elevated Ki67, implying active proliferation during primary infection (78). As time elapsed after primary infection, circulating viral-specific T cells showed decreasing proportion of cells expressing markers of activation and memory (CD38, HLA-DR, and CD45RO) and increasing proportions of cells expressing naïve and lymphocyte-homing markers (CD62L, CD57, and CD45RA) (78). Analysis of CD8+ T cells from peripheral blood and bone marrow of EBV−seropositive donors that were responsive to overlapping peptide pools of EBNA−1 and BZLF−1 identified CD45RA-/CCR7+ central memory CD8+ T cells that were capable of producing TNFα and IL-2, or TNFα only, suggesting early memory differentiation (85). In addition, the presence of CD8+ T cells specific for epitopes derived from BMLF1, BZLF1, LMP2, and EBNA3A were identified by fluorochrome-conjugated HLA class I dextramers in the lymphoid tissues of chronic EBV infection carriers, with CD69+/CD103- T cells found in the spleen and CD69+/CD103+ T cells found in the tonsils (77). In these cells, CD69 was thought to serve as a marker of tissue residence rather than T cell activation, because expression of HLA-DR in CD69+ and CD69- T cells was equal (77). Thus, distinct populations of memory CD8+ T cells may be compartmentalized in EBV carriers.

CD4+ T cell responses to EBV have also been described (76). CD4+ T cells that recognize immediate-early, early and late lytic EBV antigens isolated from PBMCs of healthy donors have been identified (86). CD4+ memory T cells responsive to latent EBV proteins EBNA1, EBNA3C, LMP1, LMP2, and BARF1 have also been identified in the blood of healthy carriers (87, 88). EBV-specific CD4+ T cell clones capable of IFNγ production in response to stimulation with peptides derived from lytic and latent EBV proteins have been successfully generated (89, 90). The frequencies of CD4+ EBV-specific T cells were lower than CD8+ T cells (88). EBNA1 specific CD4+ T cells showed a Th1 cytokine secretion profile upon stimulation with autologous dendritic cells infected with vaccinia vectors expressing EBNA1 (91). Furthermore, multiple reports indicate that CD4+ T cells that recognize various lytic or latent EBV epitopes are capable of killing EBV LCLs *in vitro* (76, 89, 92). It has been reported that LCLs express Fas and tumor necrosis factor-related apoptosis-inducing ligand (TRAIL) receptor 2 and that EBV-specific CD4+ T cells mediate LCL lysis via a combination of HLA class II-restricted recognition, Fas/Fas ligand (FasL), and the TRAIL pathways (92). EBV-specific CD4+ Th1 and Th2 cells express the cytolytic molecule granulysin, and that cytotoxicity is inhibited by oncanamycin A and ethyleneglycotetraacetic acid, which inhibit granule exocytosis, implicating granulysin as a potential mechanism of LCL killing (93). Another study demonstrated that CD4+ T cell driven cytotoxicity relied on perforin/granzyme because it was inhibited by concanamycin A (94). Interestingly, EBV micro-RNAs have been shown to inhibit Th1 differentiation of naïve CD4+ T cells co-cultured with autologous LCLs by suppressing production of IL-12 family cytokines and interfere with antigen processing and presentation via MHC class II molecules in EBV-infected B cells, thereby resulting in diminished recognition and killing of infected B cells by EBV-specific CD4+ T cells in co-culture (95). Similarly, EBV micro-RNAs help the virus to evade CD8 T cell immune responses in co-culture by inhibiting MHC class I antigen processing and presentation, reducing IFNγ production by CD8+ T cells and suppression of EBNA1 antigen gene expression, thereby potentially contributing to reduction of CD8+ T cell immune surveillance and establishment of life long EBV infection (96).

Natural killer (NK) cells become activated as part of the innate immune responses to primary EBV infection and play a role in the immediate response to acute infection and in prevention of viral tumorigenesis. As shown in a study that involved 26 patients with IM, plasma levels of NK cell-regulating cytokines IFN−α, IFN−β, and IL−12 increased, and NK cell numbers and the proportion of CD56^bright^ regulatory NK cells expanded. NK cells from infected individuals showed a heightened cytotoxic potential directed against EBV LCLs as measured by chromium release cytotoxicity assays. There was an inverse correlation between the degree of NK cell activation and level of virus detected in the blood of symptomatic patients with primary EBV infection (80, 97). Furthermore, cases of persistent EBV viremia that resulted in organ infiltration with EBV-specific T cells and renal failure in individuals whose NK cells and monocytes did not express CD16A have been described (98). NK cells from patients with complete CD16A deficiency could not mediate antibody-dependent cellular cytotoxicity, but these individuals did show a reduced “adaptive” NK cell population, suggesting that CD16A deficiency may impair antibody sensing and expansion of the “adaptive” NK cell population in response to infection (98). Remarkably, “adaptive” NK cell expansion was observed in the index patient after treatment with stem cell transplantation (98). Interestingly, CD56^bright^/CD16-NK cells, when activated by dendritic cells *in vitro*, were able to limit the proliferative potential of B cells infected with EBV in *in vitro* co-cultures; it was also noted that exogenous addition of IFNγ could also delay EBV-driven transformation, thus suggesting a possible mechanism for NK cell effect (99). In severe combined immunodeficiency (SCID) mouse models, combined GM-CSF, and low-dose IL-2 therapy helped to prevent EBV LPD in a manner dependent on the presence of human NK cells, CD8+ T cells and monocytes, while depletion of human NK cells enhanced IM-like symptoms and promoted lymphomatous tumorigenesis (100, 101). It has been observed that NKG2A-expressing NK cells derived from healthy human donors respond to autologous EBV-infected LCL in co-culture by CD107a and intracellular IFNγ staining and cytotoxic activity (102).

## EBV-Driven Immune Evasion

**FIGURE 3:**
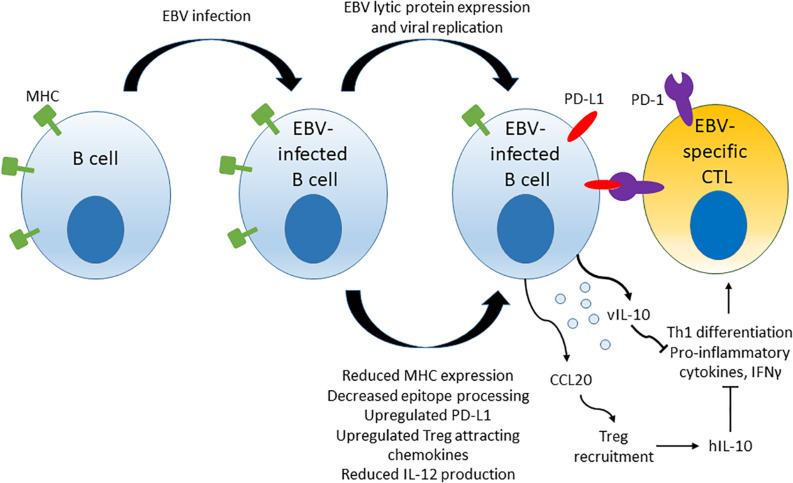
Mechanisms of T cell response evasion by EBV. EBV interferes with viral epitope processing and reduces MHC molecule expression in infected host cells, induces up-regulation of immune checkpoint molecule PD-L1 and secretes cytokines and chemoattractants that stimulate Treg recruitment and inhibiti anti-viral CD4+ T cell responses by blocking the differentiation of naïve CD4+ T cells to Th1 cells.

Epstein-Bar virus drives some of the mechanisms of immune evasion that are thought to contribute to the development of EBV LPD. Multiple viral components, including micro-RNAs, lytic and latent proteins, have been implicated in EBV-driven evasion of cytotoxic T cell responses. A model summarizing some of the mechanisms of immune evasion driven by EBV is shown in Figure 3. Mechanisms include down-regulation of viral antigen presentation in host MHC molecules, up-regulation of PD-L1 immune checkpoint molecules on infected B cells, up-regulation of chemokine attractants such as CCL20 that stimulate recruitment of Tregs to the surrounding microenvironment, reduction of IL-12 production and ultimate blockage of naïve CD4+ T cell differentiation to Th1 cells that in turn leads to reduced production of pro-inflammatory cytokines and reduced cytotoxic responses against the infected host B cells.

Several lytic phase proteins modulate immune recognition of EBV viral epitopes and adaptive immune responses by interfering with viral epitope presentation on MHC class I and class II molecules. BZLF-1 downregulates the expression of MHC class II by repression of the host transcriptional co-activator CIITA that is a major regulator of MHC class II gene expression (103, 104). BGLF-5 induces host mRNA instability, resulting in reduced synthesis of HLA class I and II molecules (105). BNLF-2a interferes with viral peptide display on MHC class I molecules by inhibiting the import of peptides into the endoplasmic reticulum via the heterodimeric ATP-binding cassette transporter complex (TAP), which interferes with pre-presentation processing of viral peptide epitopes (106, 107). BILF-1 physically associates with MHC Class I, enhances endocytosis and lysosomal degradation of surface MHC Class I molecules and impairs trafficking of newly synthesized MHC Class I molecules to cell surface in LCLs, thereby reducing the efficiency of viral epitope presentation (108, 109). BDLF-3 induces ubiquitination and reduced surface expression of MHC Class I and –II molecules (110). BDLF-3 also interferes with MHC-peptide recognition by T cells by creating a glycan shield on surface of infected cells (111). EBV viral micro-RNAs have also been implicated in immune evasion. Micro-RNAs suppress host cell surface MHC class II molecule expression, reduce the expression of lysosomal enzymes involved in antigen processing and down-regulate TAP in host cells, thus controlling epitope processing and MHC presentation (95, 96).

Furthermore, EBV directly affects the differentiation and recruitment of various T helper cell subsets to the microenvironment surrounding transformed EBV-infected B cells. EBV micro-RNAs suppress IL-12 production by infected B cells, which represses CD4+ Th1 cell differentiation and indirectly diminishes cytotoxic function of CD8+ T cells (95, 96). EBV protein BCRF1, a viral homologue of IL-10 (vIL-10), is secreted by EBV-infected B cells and reduces production of pro-inflammatory cytokines such as IFNγ by co-cultured PBMCs, suggesting reduced Th1 CD4+ T cell differentiation (112).

Multiple studies have noted an association between expansion of regulatory T cells that is associated with EBV-positive hematologic malignancies, suggesting that EBV infection may directly affect T cell populations and suppresses cytotoxic EBV-specific T cell activity in patients with EBV LPD. However, multiple studies have also shown evidence of recruitment of immunosuppressive regulatory T cells into the vicinity of EBV-positive tumor microenvironment. Tumor cells and tumor cell-derived cell lines in EBV-associated HL were found to have increased expression of the chemokine CCL20, which increased chemotaxis of immunosuppressive CD4+/CD25+ Treg cells *in vitro* (113). Analysis of T cell composition within tumor samples in 102 patients with classical HL, where 29% of the tumor samples were positive for LMP1 by immunohistochemistry, demonstrated higher numbers of CD8+ T cells and CD4+/FoxP3+ T regs in samples from EBV-positive tumors (114). Furthermore, flow cytometric analysis of T cells in peripheral blood of 45 children with B-ALL (13% with acute EBV infection and 31% with latent infection), and 40 healthy controls (10% with chronic EBV infection) in Egypt showed that patients had higher proportions of CD4+/CD25high+ Tregs, suggesting that higher Tregs may be associated with B-ALL, EBV positivity, or both (115). The study did not discern between the association of Treg prevalence in acute and chronic infection, and did not compare EBV-seropositive cancer-free subjects to B-ALL subjects, thus making it difficult to discern whether there is an association between Treg prevalence and B-ALL development in EBV-seropositive individuals (115). A study of 71 early stage ENKTL patients demonstrated that subjects with detectable pre-treatment plasma EBV DNA (53%) had higher proportion of CD4+/CD25+ Tregs in the peripheral blood and showed worse outcomes after treatment with gemcitabine, oxaliplatin and L-asparaginase/pegylated asparaginase (P-GEMOX), compared to patients that did not have detectable plasma EBV DNA pre-treatment (116).

Interestingly, the relative frequency of Tregs was noted to increase in acute HIV infection, but subsequently restored during chronic infection treated with cART, suggesting that uncontrolled HIV infection may contrubite to EBV immune escape mechanisms (117, 118). It must be noted, however, that gene expression profiling in EBV-positive HL samples demonstrated that EBV-positive samples were enriched in genes characteristic of Th1 and antiviral responses, and genes inducible by IFNγ, T cell activation and Th1 activity, suggestive of EBV-directed effector T cell responses, although presence of Th1 activity did not correlate with better outcomes (4).

Immune checkpoint molecule interactions have also been implicated in immune evasion by EBV. The latent EBV protein EBNA-1 induces up-regulation of the immune checkpoint molecule PD-L1 in latency III-expressing BL and DLBCL via repression of transcription of miR-34a (119). miR-34a is a host a micro-RNA that is induced by the tumor suppressor p53, is thought to down-regulate PD-L1 expression by interacting with the 3’UTR region of PD-L1 and has been reported as down-regulated in hematologic malignancies (120–122). The ability of PD-1/CTLA-4 blockade to inhibit EBV lymphoma proliferation in a cord blood-humanized mouse model, in which EBV-infected human cord blood is injected intraperitoneally into NSG mice, suggests that EBV-directed T cell responses may play a significant role in the control of EBV LPD (123). In this model, EBV-infected lymphoma cells expressed inhibitory ligands PD-1, PD-L1, and PD-L2, while T cells expressed PD-1 and CTLA-4 receptors (123). Combined PD-1/CTLA-4 blockade inhibited tumor growth, stimulated the response of T cells to EBV pepmix, and enhanced CD4+ and CD8+ T cell infiltration in tumors (123). Furthermore, tumor tissue analysis showed that five of five EBV DNA-positive patients and two of seven EBV DNA-negative patients showed PD-L1 expression, suggesting that T cell senescence is more prevalent in EBV-positive tumor microenviroment (116). Recently, analysis of PD-L1 expression in human NHL tumors showed that PD-L1 expression was significantly higher in EBV-positive (18/32, 56%) than EBV-negative NHL (4/38, 11%, and *p* < 0.001) (124). This finding has implications for treatment in certain clinical scenarios, since EBV-positive NHL cases showed better responses to immune checkpoint inhibition with pembrolizumab (7/15 patients with EBV positivity responded, compared to 0/15 patients with EBV-negative tumors) (124).

## T Lymphocyte Dysfunction in Acute and Chronic HIV Infection

Individuals with a functional T cell impairment are at an increased risk of developing EBV LPD, including the elderly, patients with inherited immune deficiencies and patients with iatrogenic or disease-related immune suppression, such as organ transplant recipients and sufferers of HIV. We will describe immune changes associated with HIV infection in order to highlight features that may contribute to increased susceptibility of HIV-positive patients to EBV LPD.

Human immunodeficiency virus virions preferentially infect CD4+ T cells when the Env glycoprotein binds cell surface protein CD4 and co-receptor CCR5 or CXCR4, leading to the fusion of viral and host cell membranes, entrance of the viral capsid into the cell, subsequent reverse transcription of viral RNA genome into dsDNA and integration into the host genome [reviewed in Barré-Sinoussi et al. (125)]. In acute HIV infection, HIV viremia initially drives the proliferation of CD4+ T cells, and HIV selectively infects them as they are expanding. Gut-associated CD4+ T cells are lost in several weeks after infection, followed by peripheral blood CD4+ T cells more than a year of infection (126–129).

Immune activation of CD4+ and CD8+ T cells has been a hallmark of immune response to acute HIV infection, and levels of activation marker CD38 expression correlate positively with the level of viremia and increase over time and stabilized after 8–12 weeks, while CD4+ T cell count decreases in untreated patients (128). The degree of CD4+ and CD8+ T cell activation correlates with reduced survival in patients with advanced HIV disease (130). Naïve CD45RA+/CD62L+ CD4+ and CD8+ T cell reservoir decreases over time in untreated HIV infection (131). HIV-specific CD4+ T helper proliferative responsiveness to the HIV antigen p24 in untreated patients decreases with increasing HIV viral load (132). Cytotoxic CTL activity after *in-vitro* stimulation with CD3-directed antibody, followed by the addition of autologous B-LCL infected with vaccinia virus expressing Gag, was strongly associated with the magnitude of proliferative response to p24. This indicates that CD4 help was needed for cytotoxic activity, in agreement with the report of the importance of viral-specific Th response for control of HIV viremia (132, 133). It has been hypothesized that HIV progression is associated with a switch from Th1 to the Th2 response, where Th2 response fails to protect from disease progression (134). Data on Treg responces to HIV have been conflicting. Decline in the propotions of circulating Treg and Th17 cells over time and a positive correlation between decline in Tregs, falling CD4+ counts and increase CD8+ T cells activation, measured by co-expression of CD38 and HLA-DR, has been observed in chronic, untreated HIV infection (135). Other studies, however, have reported that the proportion and absolute count of mucosal and circulating Tregs increased in untreated HIV patients (117, 136).

Human immunodeficiency virus-specific CD8+ T cells expand after HIV infection. Activation of CD8+ T cells, marked by expression of CD38, has also been reported in acute and long-term HIV infection and was found to be a predictor of progression to AIDS and mortality (130, 137–139). A study comparing PBMC samples from 98 HIV-positive patients and age-matched HIV-negative controls found that HIV-positive patients had lower CD4+ T-cell and higher CD8+ T cell counts, increased levels of immune activation as marked by soluble CD14 level and increased percentages of CD38+/HLA-DR+ markers in both CD4+ and CD8+ T cells, higher Treg cells, and higher percentage of CD4+ T cells expressing PD-1, as well as shorter telomere length (140). Interestingly, the presence of inflammatory markers correlated with shorter telomere length and increased regulatory T-cell levels (140).

Furthermore, expression of markers of T lymphocyte senescence, PD-1 and CTLA-4, has been identified in CD4+ and CD8+ T cells in chronic, untreated and treated HIV infection. A cross-sectional study of 41 untreated subjects with acute and chronic HIV infection, including elite controllers of viremia with HIV persistently lower than 50 RNA copies per ml plasma, viremic controllers with HIV levels at 50–2000 copiers per ml plasma, and patients with progressive infection showed that higher levels of CTLA-4 expression on HIV-specific CD4+ T cells positively correlated with the levels of HIV viremia and progression of infection, as well as reduced IL-2 production (141). CTLA-4 expression reduced with initiation of cART and control of viremia, but remained higher than that seen in CMV-specific CD4+ T cells in the same subjects (141). HIV-specific CD8^+^ T cells express PD-1 and upregulate genes associated with PD-1 signaling. Elevation of expression of Tim-3 and PD-1 was detected on CD4+ and CD8+ T cells early in HIV infection, and simultaneous blockade of Tim-3 and PD-1 pathways resulted in enhanced IL-2 and IFNγ production on stimulation of T cells with anti-CD3 and anti-CD28 antibodies or HIV GAG peptides, indicating that induction of T cell senescence may play an important role in HIV-driven immune evasion (142).

Chronic, treated HIV infection, in which viral load is control by antiretroviral therapy, is also accompanied by immune dysregulation despite control of viremia and improvement in CD4+ T cell counts. Elevated levels of activated CD4+ and CD8+ T cells, marked by expression of activation markers such as HLA-DR, have been reported in HIV-positive patients receiving cART, although levels of inflammatory cytokines and the numbers of activated cells decrease with initiation of therapy, particularly if therapy is initiate early during acute infection (143, 144). Depletion of naïve CD4+ T cells in patients on long term cART was also reported (145, 146). Low absolute Treg counts were also reported in patients that have been on cART for at least 1 year, and although CD4+/CD25+ T cells were hyporesponsive to stimulation with CD3- and CD28-directed antibodies and HIV and CMV antigens, they were able to suppress CD4 T-cell proliferation in response to HIV and CMV antigens (147).

We have observed that HIV status has a tremendous impact on immune reconstitution after hematopoietic stem cell transplantation in patients with hematologic malignancies (148, 149). Forty HIV-positive patients with HIV-related lymphomas that were treated with autologous stem cell transplantation on BMT-CTN-0803/AMC-071 phase II study and performed well clinically, with 2 year OS of 82% (95% CI, 65.9–91) and 2-year PFS of 79.8% (95% CI, 63.7-89.4), comparable to those of 151 matched historical control HIV-negative counterparts (150). However, HIV-positive patients demonstrated significant differences in immune reconstitution throughout the post-transplant year, including elevated absolute levels of activated T cells, cytotoxic memory and effector T cells and lower numbers of naïve and memory T helper cells (148). We also observed significantly lower numbers of activated CD19+ B cells and NK cell subsets in HIV-positive autolgous stem cell transplant recipients (148). Furthermore, when we compared immune reconstitution of HIV-positive recipients of autologous and allogeneic stem cell transplantation to HIV-negative hematopoietic stem cell transplantation and healthy control volunteers by flow-cytometry based analysis, we observed that chronic HIV infection imposed pro-inflammatory immune features on the phenotypic and functional profiling of the cellular immunome of stem cell transplant recipients, irrespective of allogeneic, or autologous stem cell donor source (149).

Multiple differences noted in the immunomes of patients with acute and chronic, untreated and treated HIV infection suggest the possibility of reduced immune surveillance against non-HIV viruses in HIV-infected subjects. HIV-infected individuals have demonstrated progressive loss of Th cell responsiveness to non-HIV viral antigens including influenza A virus, tetanus toxoid, HLA alloantigens, or phytohaemagglutinin, measured by IL-2 production or proliferation assay, indicating reduced competency of Th cells (136, 151). Reduced diversity in T cell receptor (TCR) repertoir, explored by RNA sequencing from the peripheral blood, has been noted in cART-naïve HIV-positive individuals (152). Short term use of cART did not result in restoration of TCR diversity (152). Another study showed perturbations in the TCR variable region (V) beta repertoire of naïve CD8+ T cells in HIV-infected children by following the length distribution of complementary-determining region by spectrotyping. Length distribution in CD8+/CD45RA+ but not CD8+/CD45RO+ T cells was restored after initiation of cART, suggesting a possible improvement in immune surveillance with initiation of antiretroviral therapy (153). Reduced competency of EBV-specific T cells in patients with HIV infection, compared to healthy EBV-seropositive donors, was demonstrated in a study that profiled the immune characteristics of EBV-specific T cells in 56 HIV-positive patients at different stages of HIV and in 6 HIV-positive subjects with diagnosed B-cell lymphoma (154). PBMCs derived from HIV-positive subjects with advanced HIV disease and lymphoma showed a loss of production of TNFα, IFNγ+, and IL-2 by CD4+ T cell sub-populations in EBV-stimulated cultures. CD8+ effector memory T cells were reduced in HIV-positive patients with lymphoma. Defects in cytokine production progressed in higher clinical stages of HIV infection. PBMCs of HIV-positive patients with EBV viremia showed a reduction in the TCR diversity of CD8+ T cells and decreased TNFα-producing and multifunctional TNFα+/IL-2+ and TNFα+/IFNγ+ cytotoxic T cells when cultured with EBV-positive cell lysate (154). Together, these results presented evidence of impaired CD4+ and CD8+ T cell surveillance directed against EBV in HIV-positive individuals, with exacerbated immune defects observed in advanced HIV disease and HIV-positive patients with EBV-associated lymphoma.

## Reduced EBV-Directed Immunity Places HIV-Positive Patients at a Higher Risk of EBV LPD Compared With the General Population, Despite cART

Multiple observations have suggested that EBV is more likely to cause hematologic malignancies in individuals with immune system impairments caused by HIV, iatrogenic immunosuppression, or old age, compared to the general population. EBV LPD of the elderly is a recognized clinical entity [reviewed in Castillo et al., (155)]. Post-transplant LPD is also associated with EBV in most cases (2, 6).

The frequency of EBV positivity and variety of EBV latency types associated with different lymphoma diagnoses in HIV-positive individuals were reviewed in detail elsewhere (156, 157). The prevalence of EBV-related lymphomas in HIV-positive and HIV-negative populations is summarized in Table 2. In general, for most of AIDS-defining and non-AIDS defining lymphomas, EBV association is more prevalent in HIV-positive patient cohorts. EBV positivity was reported in a 100% of 18 cases of AIDS-related primary central nervous system lymphoma, but only in 1 of 68 cases in the HIV(-) population (the positive case was HL, and there was no EBV detected in the 68 cases of primary central nervous system DLBCLs) (158, 159). DLBCL is associated with EBV in 30–90% of cases in HIV-positive cases, and 3–10% of HIV-negative cases aged >60 years (known as EBV-related lymphoma of the elderly) (5, 160–162). EBV is positive in the majority of HIV-associated Hodgkin’s lymphomas, but in HIV-negative cases there is a broad distribution of EBV positivity, from up to 40% nodular sclerosing and lymphocyte predominant types to up to 80% lymphocyte depleted and mixed cellularity types (4, 160, 163). Primary effusion lymphoma is associated with EBV (and HHV8) in more than 80% of HIV-positive cases, while HIV-negative cases are mostly reported to be EBV-negative (164, 165). EBV prevalence was reported as up to 60% in HIV-positive cases of Burkitt’s lymphoma and up to 20% in non-endemic HIV-negative cases in a multi-center study of 91 cases conducted in the United States (166).

**TABLE 2:** Prevalence of EBV positivity in sub-types of HIV-positive and HIV-negative lymphoma cases.

**Type of LPD**	**Prevalence of EBV**	
	**HIV(+) population**	**HIV(–) population**
Primary central nervous system lymphoma	100%	Mostly negative
DLBCL	30% centroblastic, 90% immunoblastic	3–10%
PEL	87% (also 100% associated with HHV8)	Mostly negative
Plasmablastic lymphoma	50%	78%
Burkitt’s lymphoma	30–60%	20% (sporadic cases)
Hodgkin’s lymphoma	80–100%	Up to 40% nodular sclerosing and lymphocyte predominant; Up to 80% lymphocyte depleted and mixed cellularity

In HIV-positive individuals, presence of EBV viremia increases the risk of a future lymphoma diagnosis. In a case control study of 49 EBV-positive and 156 EBV-negative HIV-infected patients, HIV-infected patients with EBV DNA load >100,000 IU/mL have an increased risk for diagnosis of malignant lymphoma compared to patients with EBV DNA loads ≤100,000 IU/mL. The study reported an odds ratio (OR) of 11.47 and 95% confidence interval (CI) of 4.60 for any level of detectable plasma or serum EBV, and an OR of 16.04 and 95% CI of 5.52 for EBV DNA load >100,000, as determined by real time PCR, for diagnosis of lymphoma (167). Interestingly, 52% of the HIV-related DLBCL cases identified in this study were EBV-related, similar to the general prevalence of EBV-positive AIDS-related large cell lymphomas (167, 168). Furthermore, isolation of EBV DNA from PBMCs of HIV-positive patients was associated with a greater risk of lymphoma development over the course of 3 years, and higher levels of EBV DNA were predictive of subsequent diagnosis of B cell lymphoma when measured within 1 year before the diagnosis of lymphoma (169). On the other hand, pre-treatment EBV viral load in the blood and plasma of HIV-associated HL was not predictive of clinical outcomes in patients treated with cART (170, 171).

A study of expression of EBV-encoded small RNA (EBER 1) in 128 paraffin-embedded samples of AIDS-related lymphomas showed that EBV was present in 66% of AIDS-related lymphomas. EBER 1 was detected in 100% of HL, 94% of central nervous system (CNS) NHL, 77% of immunoblast-rich/large-cell lymphomas, 34% of Burkitt-type small non-cleaved cell, and 17% of monomorphic centroblastic (diffuse large non-cleaved cell) (168). Other studies of paraffin-embedded samples of HL in HIV-positive patients found EBER1 transcript in 92–100 % of cases (172, 173). Evidence of latent EBV virus as manifested by LMP1 expression was found in HIV-associated HL (173).

## Interactions of HIV and EBV Infections Promote Lymphomagenesis

Concomitant infection with HIV and EBV may facilitate a climate for particular pathologic features of lymphomagenesis, such as accumulation of genetic lesions. Several genomic studies have suggested that EBV-positive AIDS-related lymphomas may harbor fewer genetic alterations, compared to EBV-negative lymphomas. Comparative genomic hybridization studies of 28 AIDS-related lymphomas demonstrated that DNA copy number change was less frequently associated with EBV-positive latency type II/III than in EBV-positive latency I or EBV-negative cases (174). Genome-wide single nucleotide polymorphism-based microarray comparative genomic hybridization performed in 57 HIV-related NHL and 105 HIV-negative DLBCL clinical samples and cell lines found that while HIV-positive and HIV-negative samples had a similar degree of genomic instability, genomic complexity correlated positively with EBV infection status, EBV-positive HIV-DLBCL, and HIV-PCNSL showed a lower genomic complexity than EBV-negative cases (175). HIV status did correlate with specific DNA lesions, such as deletion of fragile sites and inactivation of tumor suppressor genes FHIT, WWOX, DCC, and PARK2 (176). Greater genomic stability of EBV-positive HIV-related NHL may be related to a cooperation between the transformative effect of EBV and a reduced EBV-directed immune capacity secondary to chronic HIV infection (177).

Expression of latent EBV proteins and host B cell genes also appears to be affected by HIV status of lymphoma cases. All EBV viral latency types have been observed during a study of 94 AIDS-related lymphomas by EBER in-situ hybridization and immunohistochemistry for LMP1 and EBNA2, with exceptions including 100% plasmablastic lymphoma cases, 90% of BL and 100% of unclassifiable cases showing restriction to latency type I (178). Interestingly, the majority of AIDS-associated GCB DLBCL samples used in this post-cART era study demonstrated EBV latency type I, in contrast to previously reported findings of predominant latency type III in EBV-positive AIDS-related large cell lymphomas pre-cART (179). Analysis of host gene expression in HIV-positive and HIV-negative DLBCL samples showed significant differences, suggesting that EBV, and/or HIV may impose specific protein expression signature on host malignant cells and cells involved in immune surveillance (178). The authors speculate that this change in EBV latency type may reflect the effects of changes in the immune surveillance landscape of HIV-positive, cART-treated host, on the oncogenic mechanisms employed by the EBV virus (178).

B cell activation has been observed in chronic HIV infection [reviewed in Moir and Fauci (180)]. EBV may directly affect the transcription of cellular genes to the effect of maintating cellular transformation. BLIMP1 is a cellular transcription factor that plays an active role in B cell differentiation to plasma and has been found to act as a tumor suppressor in DLBCL (181–183). BLIMP1 expression is down-regulated in EBV-transformed GC B cells (183). BLIMP1 induces a BZLF1- and BRLF1-dependent latent to lytic switch in type III but not type I latency B cell lines and epithelial cell lines derived from gastric cancers, nasopharyngeal carcinomas, and normal oral keratinocytes (183, 184). BLIMP1 activity was found to be down-regulated by LMP1, resulting in reversal of the effect of BLIMP1 on some of its target genes, for example up-regulation of MYC that is repressed by BLIPM1, and a shutdown of viral replication (183). In a number of EBV-positive NHL lymphoma samples that included AIDS-related NHL, the promoter and exon 1 of BLIMP1 were found to be hypermethylated, which resulted in down-regulation of transcription (185). Increased expression of BLIMP1 and CD30, and reduced expression of transcription factors BCL6 and LMO2, was associated with EBV positivity in AIDS-related lymphomas on immunohistochemical staining (186). Together these data demonstrate an example of the co-optation of the cellular machinery by EBV viral proteins, thereby contributing to B cell transformation and EBV LPD development that is observed in HIV-positive individuals.

## HIV and EBV+ Lymphomas in Sub-Saharan Africa

According to the recent UNAIDS report, of the total 37.9 million people living with HIV, 25.6 million live in sub-Saharan Africa (20.6 million in Eastern and Southern Africa and 5 million in Western and central Africa) (187). The regions alone accounts for about two third of the people that ultimately die as a consequence of HIV/AIDS-related diseases.

The impact of HIV infection on the disease incidence of EBV-associated LPDs is less clear. High plasma EBV DNA level was identified as a poor prognostic factor associated with reduced OS in HIV-associated, EBV-negative DLBCL in Malawi (188). However, diagnosis of EBV-association via in-situ-hybridization for EBER is not a common practice in most countries in the Africa continent and there is a limitation on the cancer registry data. Nevertheless, recent data from the Addis Ababa Cancer registry in Ethiopia has indicated that NHL is the second most common cancer in Ethiopian men, a striking contrast to that encountered in North America where NHL is the 8th most common type of cancer in men (189). It is possible that the high rate of NHL in Ethiopia is due to the high prevalence of HIV infection in the Ethiopian male population and to oncogenic viruses such as EBV.

African populations are at risk of co-infections with EBV and other pathogens, and co-infections are seen in cases of EBV LPD. In Kenya, endemic BL associated with EBV and *Plasmodium falciparum* co-infection is one of the most common pediatric cancer (190, 191). In Uganda BL accounts for 90% of NHL children cases in the country (192). In a study of the children BL cases from Uganda Cancer Institute Record revealed that of children with BL, nearly one-third of cases were also HIV positive (193). BL in HIV positive children presented with significantly advanced disease. HIV positive children were more likely to have extrafacial disease, hepatic and thoracic masses and had poor overall survival compare to HIV negative individual (193). A study in Rwanda has indicated that HIV is associated with increased risk of infection related cancer including HL and NHL and accounting for significant proportion of referral cancer patients (194). The population most affected by these infections related cancer are children.

Treatment of cancer and AIDS-related malignancy in well-resourced centers demonstrates excellent outcomes. For example, a prospective study of treatment of plasmablastic lymphoma with intensive chemotherapy in Malawi, performed with support of the AIDS Malignancy Consortium, that involved a total of 12 patients, including 3 patients with HIV and EBER-positive disease, showed 1 year OS was 56% (95% CI 24–79%), without clear difference between HIV-positive, and HIV-negative cases, indicating that curative intent is reasonable in these clinical scenarios (195). Thus, it is time to mobilize resource and conduct clinical trials that considers the genetics, other co-infections (e.g., EBV, *P. falciparum* malaria) in the region and develop better management of lymphoma in areas of limited resource settings and unique patient populations, such as Sub-Saharan Africa.

## Conclusion and Future Directions

Epstein-Bar virus maintains a lifelong infection in B-lymphocytes in more than 90% of the world’s population. In HIV-positive individuals, the risk of EBV-positive NHL remains elevated in the cART era, despite effective control of viremia and improvement in CD4 T cell counts. A combination of EBV-related transformative factors and decreased effectiveness of immune surveillance contributes to the continued elevation of the risk of EBV LPD in the HIV-positive population.

Effots to drive development of preventive and therapeutic strategies for EBV LPD have explored virus-directed and immunomodulatory approaches targeting viral proteins, such as adoptive transfer of EBV-specific T cells that can be isolated from third party donors or generated by stimulation with viral antigens [reviewed in Cohen (196), Houghtelin and Bollard (197) and summarized in Table 3]. EBV-specific CD19 chimeric antigen receptor (CAR) T cells are also being explored, and CAR T cells directed against HLA-DR have successfully killed EBV LCLs *in vitro* (198, 199). Additional strategic approaches may include inducing a latent-to-lytic switch in the EBV infection state that is hypothesized to induce heightened immune detection and destruction of EBV-infected cells and has been shown feasible in a phase I trial targeting relapsed/refractory EBV LPD in HIV-positive patients treated with a combination of proteasome inhibitor bortezomib and ifosfamide, carboplatin, and etoposide with or without rituximab, and successfully induced an increase in EBV viral load in treated subjects (200). Another potentially useful approach could be anticipatory vaccination of high risk immunodeficient populations with a T cell response-inducing vaccine comprized of an EBV immunogenic antigen, such as BZLF1, as responses to BZLF1 have been associated with recovery from EBV PTLD (201). Vaccination of humanized PBL-SCID mouse model of EBV LPD with BZLF1-transduced dendritic cells induced specific T cell responses and prolonged mouse survival (202). Fusion protein versions of the BZLF1 viral target protein for optimization of T cell immune responses are currently being tested (203).

**TABLE 3:** Summary of EBV-directed and immunomodulatory therapies for EBV LPD.

**Preventive strategies**	
EBV vaccine development	Recombinant gp350 membrane antigen vaccine induces neutralizing antibody responses in up to 60% of subjects (206–208)
**Treatment strategies**	
EBV-directed therapy	Induction of EBV lytic cycle sensitizes EVB LPD to antiviral agents in pre-clinical anemia models, cases of primary central nervous system lymphoma (OR 92%) (209, 210), systemic HL, and NHL (OR 67%) (211, 212)
Immuno-therapeutic	Third Party EBV-specific CTL have achieved durable responses in 60% of patients with EBV LPD including post-transplant lymphoproliferative disease (OR up to 70%) (213, 214)
	Latent protein-specific CTLs that are active against EBV can be expanded and can achieve responses in type 2 latency HL and NHL (OR up to 60%) (215–218)
	Cytokine therapy with low dose interleukin-2 results in expansion of immune effectors and Th1 responses patients with HIV-associated malignancies (219, 220)

Remarkably, it has been recently reported that brentuximab vedotin (BV) may contribute to the restoration of CD4 and CD8 T cell counts in HIV-positive patients with HL (204). BV is an anti-CD30 antibody conjugated to monomethyl aurestatin, a microtubule inhibitor that selectively induces apoptosis of CD30+ cells. A combination of BV with doxorubicin, vinblastine, and dacarbazine (AVD) has been approved by the Federal Drug Administration for treatment of advanced stage HL after the Echelon-1 trial that demonstrated superior modified progression free survival in HIV-negative patients at 2 years (82%), compared to traditional treatment with doxorubicin, bleomycin, vinblastine, and AVD (77%) (205). The AMC-085/NCT01771107 phase 2 trial investigated the combination of BV and AVD in previously untreated HIV-associated, Stage II-IV HL with encouraging results of good tolerability and 2-year PFS of 86% (204). An unexpected rise in CD4+ and CD8+ T cell counts of HIV-positive patients that received BV-AVD was observed (204). Rise in T cell counts correlated with a reduction in HIV viral loads, suggesting that the increase in T cell counts is associated with an enhancement of HIV-directed T cell responses. These findings suggest that immunomodulatory therapy may help to restore immune surveillance in HIV-positive patients on cART, and may be explored in the future for potential to lower the risks of virus-associated malignancies in HIV-positive individuals with controlled HIV viremia.

Further research into the incidence, B cell immortalization mechanisms, viral latency types, and EBV-specific T cell and NK cell profiling in populations that suffer high exposure to potential co-infection with HIV, EBV and other pathogens, such as malaria, may shed additional light onto the interplay of viral immortalization and immune surveillance defects that result in increased risks of virus-related malignancies. Continued study of the impact of immunomodulatory chemo-immunotherapy targeted at the biology of EBV-induced B cell transformation and enhancement of the effectiveness of the immune response is necessary to optimize our approaches to treating EBV LPD in immunosuppressed populations, such as HIV-positive patients.

## Author Contributions

PS reviewed the literature, wrote the manuscript, and created illustrations. EA and TA reviewed the literature and added to the manuscript. AM reviewed and summarized the literature and added to the illustrations. RB reviewed the literature, edited the manuscript, and provided conceptual guidance. AM contributed to the conception of the manuscript, provided critically important revisions to the work for important intellectual content, and approved the final version of the manuscript. AM agreed to be accountable for all aspects of the work in ensuring that questions related to the accuracy or integrity of any part or integrity of any part of the work are appropriately investigated and resolved. All authors contributed to the article and approved the submitted version.

## Conflict of Interest

The authors declare that the research was conducted in the absence of any commercial or financial relationships that could be construed as a potential conflict of interest.
